# Recruitment for ADRD Clinical Trials Optimized through Academic and Community Partnership in Appalachia

**DOI:** 10.1002/alz.085812

**Published:** 2025-01-09

**Authors:** Elizabeth K. Rhodus, David Gross, Vickie Fairchild, Laura Henley, Celeste Roberts, Gregory A Jicha, Richard J Kryscio, Allison Gibson

**Affiliations:** ^1^ University of Kentucky, Lexington, KY USA; ^2^ St. Claire Healthcare, Morehead, KY USA; ^3^ University of Kentucky Sanders‐Brown Center on Aging, Lexington, KY USA; ^4^ Sanders‐Brown Center on Aging, Lexington, KY USA; ^5^ St. Louis University, St. Louis, MO USA

## Abstract

**Introduction:**

The United States is undergoing a demographic shift with increasing proportions of older adults. Currently, one in three older adults pass away with a form of Alzheimer’s disease or related dementias (ADRD). This figure is higher in underrepresented and underserved groups including older adults in rural Appalachian communities. Programs that promote inclusion in clinical services and research are needed in rural, medically underserved regions. To establish and evaluate mechanisms for outreach and recruitment in clinical research for caregivers of persons with ADRD living in rural Kentucky counties.

**Methods:**

The University of Kentucky (UK) Sanders‐Brown Center on Aging partnered with St. Claire Healthcare and the Northeast Kentucky Area Health Education Center to promote rural‐tailored clinical trial recruitment of caregivers of older adults with ADRD. Successful approaches and methods were tracked and documented.

**Results:**

A model for rural recruitment and outreach for older adult engagement emerged. Over the course of seven months, 106 rural Kentucky caregivers of persons with ADRD were screened for clinical trial enrollment. This model identified successful outlets for recruitment dissemination through multiple strategies, including community agencies, healthcare providers, social media, emailing, word of mouth, and in‐person promotion of the study (health fairs, self‐initiated conversation at community events, etc.). Flexible engagement efforts were effective, such as calling at convenient times for participants and adapting to schedule changes (i.e., changes during and following historic flooding in recruitment regions).

**Conclusions:**

Academic‐community partnerships extend the enterprise of university‐based clinical research to offer innovative programming directly in underserved and underrepresented rural communities. Recommendations from this program include 1) involving community representatives in recruitment and outreach efforts, 2) targeting communication with local organizations, and 3) including a variety in modes of contact with potential participants. This program illustrates a model that optimizes inclusion of underrepresented populations in clinical research and may have applicability to improve clinical care options in underserved areas.

